# The Way to a Generalist Herbivore's Heart Is Through Its Chemosensory Receptors: Using Multiple Approaches to Map the Genetic Basis of Adaptation in a Non‐Model Organism

**DOI:** 10.1111/mec.70017

**Published:** 2025-07-08

**Authors:** Tiago da Silva Ribeiro, Rodrigo Cogni

**Affiliations:** ^1^ Department of Ecology University of São Paulo São Paulo Brazil

**Keywords:** adaptation, experimental evolution, genomics/proteomics, quantitative genetics

How does a generalist herbivore adapt to a challenging food source? In this issue of Molecular Ecology, Villacis‐Perez et al. (2025) set out to answer this question. This is a question about the genetic basis of adaptation, a broader task that has driven both theoretical and empirical work in evolutionary biology (e.g., Elena and Lenski 2003; Johnson 2018; Bomblies and Peichel 2022). With the advance of—omics tools, these questions have started to be asked in more diverse and ecologically interesting systems, such as the *Tetranychus urticae* mite, the generalist herbivore studied by Villacis‐Perez et al. (2025). Through both experimental evolution and F2 backcrossing approaches, the authors identified genomic regions that underlie adaptation to, and reproductive performance on, a challenging host: tomatoes. Chemosensory receptor genes were identified in both approaches, suggesting a shared path between host adaptation and general increased reproductive rate. The authors offer two promising candidate genes for future research on this topic, the genes TuGR and TuENaC63. Moreover, detoxification and nutrient acquisition‐related genes are also prominent in the list of candidate genes and might underlie the adaptation to a challenging host by a generalist species as well, highlighting that host specialisation involves multiple biological functions. Interactions between herbivores and host plants have been central to our understanding of coevolution since the seminal paper by Ehrlich and Raven (1964). Today, we have the tools to investigate the genetic mechanisms shaping these interactions (Cogni et al. 2022). The paper by Villacis‐Perez et al. (2025) is a fascinating example of how multiple genomic approaches can complement each other and reveal the genetic architecture of herbivore specialisation on a host plant.

To identify the genetic underpinnings of traits of interest, samples used must contain enough heritable diversity. Herein, first, the authors measured phenotypic diversity in the parental lineages and found heritable variation among them. In their study, Villacis‐Perez et al. (2025) not only measured relevant fitness‐related traits in all lineages used but also employed two different genetic mapping strategies to strike a balance between high starting genetic diversity and high numbers of phenotyped individuals. High starting genetic variation resulted from creating a population named “Mix” by crossing six isofemale lines of *T. urticae* from different localities in Europe (Figure 1). The Mix population was subjected to 20 generations of selection on tomatoes, a challenging host to the mites. However, it is important to note that one of the isofemale lines (PL, from Poland) was field‐collected on tomatoes; this choice could have been made to include adaptive natural variation in the Mix, but it would be interesting to see results solely from lines not previously exposed to the selected host. At the end of the experimental evolution, genomic and transcriptomic data from several replicates and control populations were obtained; transcriptomic data included selected (tomato) and control (bean) mites that were exposed to bean and tomato feeding treatments before sequencing. The overall design worked to capture both allele frequency and gene expression differences that evolved as a result of experimental evolution. The high numbers of phenotyped individuals came from their second experiment: the F2 backcross. This experiment aimed at mapping the genetic basis of reproductive output (a fitness‐related trait) in a mapping population created by crossing two strains: one field collected on tomatoes and the other developed in the laboratory. Then, the F1 hybrids were backcrossed to the laboratory strain, and the F2 offspring were separated into the 15% highest and lowest performers based on the phenotyping of nearly 1000 individuals. The authors also sequenced a random control (non‐phenotyped) F2 sample and then mapped the genetic variation underlying both the high and the low reproductive performance by comparing the extreme pools to the control sample. This approach is very powerful for using phenotype measures of individual mites to create the extreme pools and for targeting natural genetic variation from a line collected on tomatoes, which was likely already the result of natural, not experimental, evolution. Lastly, comparing both approaches—one with higher starting diversity exposed to lab selection and the other targeting natural variation—has the potential to greatly elucidate the genetic basis of host adaptation, as demonstrated by the authors' results.

**FIGURE 1:**
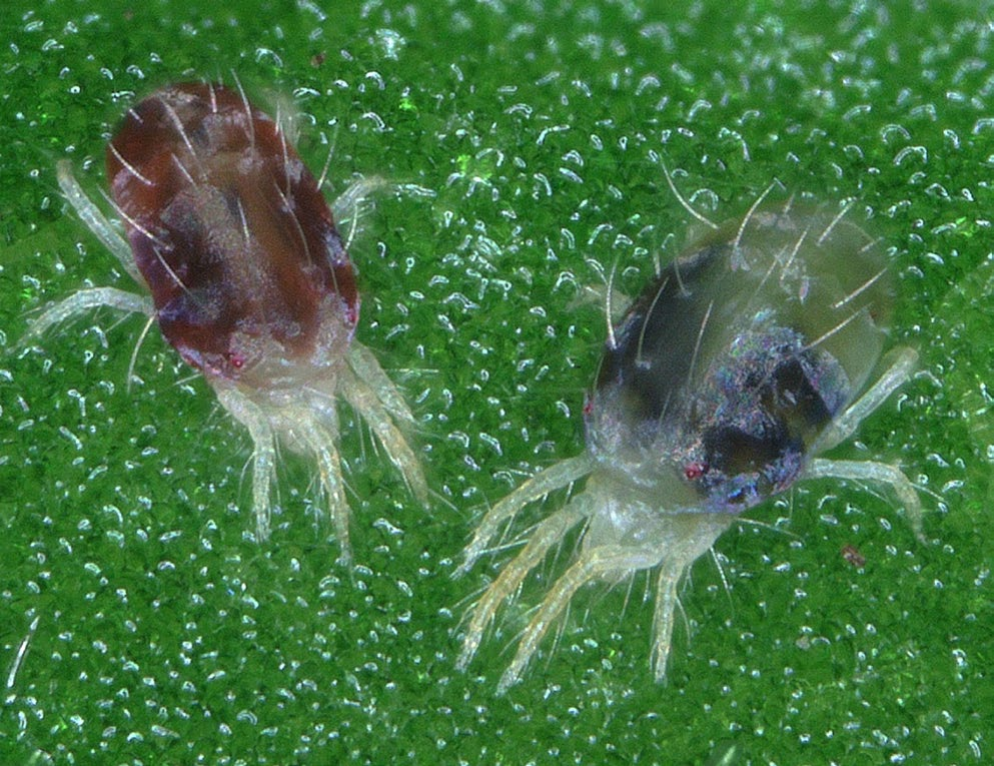
The two‐spotted spider mite *Tetranychus urticae* has an immense adaptive potential, exploiting thousands of agricultural, ornamental, and wild plant species as hosts. Photo credit: Sander De Rouck, from the Department of Plants and Crops in UGent, Belgium.

Both experiments detected many genomic regions associated with tomato adaptation and performance, which we will broadly call Quantitative Trait Loci (QTLs). Many of the QTLs were unique to each approach, which was expected given the different strategies, starting parental lines, and focus of each experiment. Nonetheless, the region with the strongest association found for high reproductive performance in the F2 experiment overlaps with a QTL detected for tomato adaptation in the experimental evolution. Interestingly, the genes in this region include gustatory chemosensory receptor genes (TuGR). It is straightforward to envision how gustatory receptor genes could play a role in host adaptation to a new feeding source. Interestingly, changes in these genes might also have led to high reproductive performance, suggesting that the increased performance may result from selected populations better recognising the new host as a food source and therefore getting more energy and nutrition.

The different experiments captured unique adaptive variants that did not overlap between the distinct designs. Given that the two experiments used unique parental lines, these results suggest the existence of common adaptive variants in the natural populations. This claim is also supported by (1) the identity of experimental evolution QTLs tracking to different parental strains and not only to the one collected on tomatoes and (2) the broad genetic differences of the selected replicates shown by the PCA results. The PCA of the selected Mix replicates showed a wider genetic spread than the control, which can be attributed to stronger genetic drift when exposed to a challenging environment but could also merit further investigation of how much the adaptive routes of each replicate diverged from each other. For example, it would be interesting to see if the unique outliers from each replicate are enriched for similar biological functions and pathways involved in host adaptation.

The outcomes of the selection experiment were also studied in terms of the evolution of differences in gene expression in mites feeding on beans and tomatoes. The transcriptome data was analysed, focusing on two groups of genes: group 1 had genes differentially expressed (DE) between experimental treatments (tomato‐selected vs. control) when raised on bean leaves, and group 2 had genes in which differential expression was significant based on the interaction of both selection treatment and the feeding treatment (raised on bean vs. tomato leaves). This interesting approach allows one to mine the list of genes within QTL regions for genes that are also DE and create lists of candidate genes for downstream analyses based on multiple evidence of heritable and functional roles underlying host adaptation. The strongest candidate genes were related to taste perception, detoxification, and nutrient acquisition. Many genes that showed evolved differential expression patterns also showed a plastic expression response when first exposed to tomatoes, but the majority of the selected DE genes did not show an initial response. Additionally, whether DE genes were found in or out of QTL regions was used to indicate cis‐ or trans‐regulatory evolution patterns (see Ji et al. 2023 for a study on trans‐regulated differences in this species). However, we recommend caution when comparing lists of DE genes with each other, as well as with QTL regions. The presence or absence of genes in these lists can be influenced by differences in the statistical power of different analyses.

Understanding the genetic basis of adaptive traits is a key challenge to quantitative and evolutionary genetics, with approaches ranging from GWAS in natural populations to artificially constructed laboratory populations. The different approaches have unique strengths and weaknesses (Complex Trait Consortium 2003), and combining them helps us paint a bigger picture of the adaptive process. The results from the experiments performed by Villacis‐Perez et al. (2025) presented interesting lists of candidate genes and biological functions underlying host adaptation in this generalist species, and their complementary nature also offered us insights into how adaptation and reproductive performance might have a shared genetic basis.

## Author Contributions

T.S.R. and R.C. contributed to the writing and discussion of this article.

## Conflicts of Interest

The authors declare no conflicts of interest.

## Data Availability

The authors have nothing to report.

## References

[mec70017-bib-0001] Bomblies, K. , and C. L. Peichel . 2022. “Genetics of Adaptation.” Proceedings of the National Academy of Sciences of the United States of America 119, no. 30: e2122152119. 35858399 10.1073/pnas.2122152119 PMC9335183

[mec70017-bib-0002] Cogni, R. , T. B. Quental , and P. R. Guimarães Jr. 2022. “Ehrlich and Raven Escape and Radiate Coevolution Hypothesis at Different Levels of Organization: Past and Future Perspectives.” Evolution 76, no. 6: 1108–1123. 35262199 10.1111/evo.14456

[mec70017-bib-0003] Complex Trait Consortium . 2003. “The Nature and Identification of Quantitative Trait Loci: A Community's View.” Nature Reviews Genetics 4, no. 11: 911–916. 10.1038/nrg1206 PMC2063446 14634638

[mec70017-bib-0004] Ehrlich, P. R. , and P. H. Raven . 1964. “Butterflies and Plants: A Study in Coevolution.” Evolution 18: 586–608.

[mec70017-bib-0005] Elena, S. F. , and R. E. Lenski . 2003. “Evolution Experiments With Microorganisms: The Dynamics and Genetic Bases of Adaptation.” Nature Reviews Genetics 4, no. 6: 457–469. 10.1038/nrg1088 12776215

[mec70017-bib-0006] Ji, M. , M. Vandenhole , B. De Beer , et al. 2023. “A Nuclear Receptor HR96‐Related Gene Underlies Large Trans‐Driven Differences in Detoxification Gene Expression in a Generalist Herbivore.” Nature Communications 14, no. 1: 4990. 10.1038/s41467-023-40778-w PMC10435515 37591878

[mec70017-bib-0007] Johnson, M. 2018. “Integrating Selection Mapping With Genetic Mapping and Functional Genomics.” Frontiers in Genetics 9: 603. 30619447 10.3389/fgene.2018.00603 PMC6295561

[mec70017-bib-0008] Villacis‐Perez, E. , F. De Graeve , B. De Beer , et al. 2025. “Independent Genetic Mapping Experiments Identify Diverse Molecular Determinants of Host Adaptation in a Generalist Herbivore.” Molecular Ecology: e17618. 39676612 10.1111/mec.17618

